# Beneficial effects of intermittent intravenous saline infusion in dysautonomic patients with Myalgic Encephalomyelitis/Chronic Fatigue Syndrome: a case-series

**DOI:** 10.3389/fneur.2025.1601599

**Published:** 2025-07-21

**Authors:** Per Sjögren, Helena Huhmar, Bo C. Bertilson, Björn Bragée, Olli Polo

**Affiliations:** ^1^Bragée Clinics, Stockholm, Sweden; ^2^Department of Neurobiology, Care Sciences and Society, Karolinska Institutet, Huddinge, Sweden

**Keywords:** intermittent infusion, volume loading, ME/CFS, treatment, dysautonomia

## Abstract

**Purpose:**

Myalgic Encephalomyelitis/Chronic Fatigue Syndrome (ME/CFS) is a debilitating condition with no single, uniformly effective pharmacologic therapy. Dysautonomic features like orthostatic intolerance and postural tachycardia syndrome are common features in ME/CFS, severely affecting the patient’s quality-of-life. Intermittent saline infusion may reduce symptoms associated with dysautonomia, but this has not been tested scientifically in patients with ME/CFS.

**Methods:**

In this case-series, 22 patients with ME/CFS and signs of dysautonomia and/or hypovolemia were treated every third week over 9 weeks with intravenous saline (9 mg/mL NaCl), using standard aseptic technique. Symptoms were monitored throughout the treatment regime, and a follow-up evaluation was conducted.

**Results:**

At treatment start, patients were predominantly female (95%), at mean age 46 ± 10 years, and with a mean body hydration percentage of 48 ± 6. Self-reported health status revealed an overall symptom score of 47 ± 13 on a 0–96 scale, a median POTS score of 64 (IQR 16) on a 0–120 scale, and poor measures of quality-of-life (median 25 IQR 25, on a 0–100 scale) and ability-to-work (median 0, IQR 26, on a 0–100 scale). Following 9 weeks of intermittent saline infusion (mean volume 1,600 ± 360 mL), self-reported composite symptom score, quality-of-life and POTS-related symptoms improved significantly (all *p* < 0.001), as did ability-to-work (*p* < 0.05).

**Conclusion:**

Our data derived from a non-controlled case-series indicate health benefits from volume loading with intermittent infusion of saline among patients with ME/CFS, which may stimulate further studies on various forms of intravenous volume loading to patients with ME/CFS and dysautonomia.

## Introduction

Myalgic Encephalomyelitis/Chronic Fatigue Syndrome (ME/CFS) is a debilitating condition with unknown pathophysiology and lack of any single, uniformly effective pharmacologic therapy. The estimated prevalence of ME/CFS ranges between 0.5 and 2% in the population (1, 2), and is associated with an enormous economic burden (2). The disease presents with a wide range of neurological, autonomic, gastrointestinal and/or immunological symptoms including fatigue, pain, and sleep disturbance. The presence of post-exertional malaise (PEM) is required for diagnosis (3, 4). Dysautonomic syndromes like classical and delayed orthostatic hypotension, neurally mediated hypotension, postural tachycardia syndrome (POTS), and orthostatic intolerance (OI) in the absence of heart rate and blood pressure changes are common in ME/CFS and can severely affect the patient’s daily life (5, 6). POTS is defined as a symptomatic (dizziness, palpitation) increase of heart rate ≥30 beats/min without a drop in blood pressure when standing up from the lying position (7). Hypovolemia and venous pooling have been identified as contributing factors to POTS in patients with no clear comorbidities (8–12). A similar scenario with hypovolemia and preload failure has been described in ME/CFS-patients with POTS (13–15), suggesting a potential therapeutic pathway in ME/CFS.

There are currently no uniformly effective treatments for ME/CFS. However, many hypovolemic patients with ME/CFS and OI could be treated according to the existing guidelines for orthostatic hypotension or POTS (7). Correcting hypovolemia with intravenous (IV) saline infusion represents one treatment strategy to counteract OI in ME/CFS. Several studies have documented instant improvement of hemodynamics and OI after single infusions of IV saline (16–20). As there is not enough evidence for the safety of repeated IV-saline infusions in POTS, the international consensus guidelines from 2015 do not recommend it for long-term treatment (7). In the study by Ruzieh et al., though, intermittent infusions of saline dramatically reduced symptoms and improved quality of life in patients suffering from refractory POTS (21). In view of the severely compromised quality of life without effective symptom relief in ME/CFS, the short and long-term efficacy and safety of intermittent saline infusion deserves to be studied.

We report here a clinical series of intermittent saline infusion over 9 weeks in 22 ME/CFS patients with signs of dysautonomia and POTS.

## Patients and methods

### Participants

Patients selected for this treatment were recruited internally at the Bragée ME center (Stockholm, Sweden), with established ME/CFS diagnoses according to the Canadian Consensus Criteria (4). Clinical routines at Bragée ME center included a standardized head-up-tilt test to assess OI or POTS. Forty (40) patients with signs of dysautonomia, POTS and/or hypovolemia during diagnostic assessment, or at clinical follow-up, were considered having clinical indications for IV saline therapy. Eighteen out of 40 individuals were treated only once or twice and did thus not complete the full treatment regime: the reasons for not completing the treatment were predominantly (i) sudden illness preventing attendance at certain treatment session or (ii) due to the effort related to the treatment procedure. Data presented herein are focused on those 22 patients who completed the treatment regime over 9 weeks. Ethical approval of the project was given by the Swedish Ethical Review Board (no. 2021-03114 and no. 2022-01471-02) and written informed consent was obtained from all participants prior to treatment start.

### Infusion protocol

The IV infusion of physiological saline solution was performed by standard aseptic technique using saline (Braun, 9 mg/mL NaCl) during a 3 h-session and administrated at room temperature, starting with a slow drip rate with subsequent adjustments to achieve a final infusion volume of 1,000–2000 mL, and repeated every third week over 9 weeks (i.e., a maximum of 3 infusions) with subsequent follow-up.

### Examination of orthostatic intolerance

OI was assessed with an electronic tilt table during 10 min of tilting at 80 degrees. Prior to tilting, patients remained in supine position for at least 5 min while blood pressure and heart rate measurements were monitored to confirm hemodynamic stability. POTS was defined as a sustained increase in heart rate of ≥30 beats per minute, accompanied by characteristic orthostatic symptoms in the absence of postural hypotension. This abbreviated procedure is consistent with the diagnostic criteria outlined by Freeman et al. (22), and was selected to allow standardized yet clinically feasible assessment in a non-cardiology outpatient setting.

### Measurements and follow-up

Prior to each treatment session, patients had a brief clinical check-up with a specialist doctor (HH, OP) to update their overall health status. In addition, information about heart rate, blood pressure, and blood saturation were collected by a nurse before, during and after each infusion session. Before and after each infusion, anthropometrics were collected and body composition determined by bioelectrical impedance analysis (BIA), using Tanita MC-980MA (Tanita Corporation, Tokyo, Japan) by participants standing on the scale lightly dressed and barefooted following emptying of the bladder. A body hydration percentage between 45 and 60 was considered normal for women and 50–65 for men. An additional TILT-table test was carried out in 17 of the patients within a two-week period following end-of-treatment (mean 9.2 days). A standardized questionnaire including the 24 most prevalent symptoms associated with ME/CFS was provided to every patient prior to each treatment session. The symptoms included joint pain, body ache, morning stiffness, attention deficits, memory difficulties, brain fog, generalized pain, sensitivity to light/sound, headache/migraine, urinary incontinence, polyuria, upset stomach, numbness/prickly sensations, coordination difficulties, dizziness, tender lymph nodes, sore throat, feeling feverish, palpitations, flu-like symptoms, fatigue, and sleeping problems. The intensity of their symptoms was reported as mild, moderate, severe, or unbearable. All 22 patients completed the questionnaire in relation to the first treatment session, while 21 patients completed the questionnaire in relation to second and third treatment sessions. A composite symptom score was derived from the questionnaire using the following algorithm: Overall symptom score = number of symptoms with mild severity + (number of symptoms with moderate severity *2) + (number of symptoms with severe severity *3) + (number of symptoms with unbearable severity *4). Thus, the composite symptom score could range from a minimum of 0 to a maximum of 96.

Also, 1 week after the third and final treatment session, patients were asked to complete a simple follow-up questionnaire including subjective measures on treatment effects including: (1) standardized symptom questionnaire (as described above); (2) benefit of treatment (Likert scale 0–10); (3) effort of the treatment regime (Likert scale 0–10); (4) experiencing PEM or other side-effects of the treatment; (5) desire to continue the treatment (no/maybe/yes); (6) working ability before and after treatment (0–100%); (7) QoL before and after treatment (0–100%); and (8) free-text comment. Also, the Malmo POTS Questionnaire was provided at follow-up to capture symptoms of dysautonomia/POTS before and after treatment. The Malmo POTS Questionnaire covers 12 typical symptoms of dysautonomia/POTS with a Likert-scale 0–10 for each symptom, yielding a maximum total score of 120 (23). The follow-up was completed by 19 patients.

### Statistical analysis

MS-Excel (Microsoft, Redmond, Washington) and Statistics Kingdom (statskingdom.com, an on-line statistical resource, Melbourne, Australia) were used for data processing. Normally distributed data are presented as mean (SD), otherwise as median (IQR). Welch’s *t*-test or Mann–Whitney U test were used for group comparison at baseline, Wilcoxon signed rank tests performed to analyze changes from pre- to post-treatment, and repeated measures ANOVA to test for changes in repeated measures over the treatment period (i.e., BP, DBP and SBP).

## Results

Baseline characteristics of patients with ME/CFS treated with IV saline are presented in Table 1. Twenty-two (22) patients completed all three treatments over 9 weeks, with a mean body hydration percentage of 48 ± 6. The average disease duration prior to treatment was 32 ± 19 months, ranging from mild to severe. The self-reported health status at treatment start was captured by standardized questions revealing poor QoL, (median 25, on a 0–100 scale with 100 reflecting the best possible QoL), low working capacity (median 0, on a 0–100 scale with 100 reflecting full working capacity) and with a mean composite symptom score of 47 (min 27, max 70), that could range between 0 and 96, with 96 reflecting worst possible overall symptoms. Baseline characteristics of those who did not fulfill the treatment regime (non-completers *n* = 18, i.e., only received IV saline once or twice) did not deviate significantly from completers as shown in Table 1 (all *p* > 0.20), except for BMI being significantly higher in non-completers (*p* < 0.001).

**Table 1:** Characteristics of patients with ME/CFS prior to treatment start, categorized into those who completed, and not completed, intermittent infusion with intravenous saline over 9 weeks.

Characteristics	Completers of the treatment (*n* = 22)	Non-completers of the treatment (*n* = 18)
Age in years, mean ± SD	46 ± 10	48 ± 9
Female gender, *n* (%)	21 (95)	15 (83)
BMI kg/m^2^, mean ± SD	24.9 ± 4.7	27.3 ± 6.8^*^
Disease duration in mo., mean ± SD	32 ± 19	30 ± 15
Disease severity ^a^, *n*		
Mild	2	0
Mild-to-moderate	2	1
Moderate	7	12
Moderate-to-severe	3	3
Severe	5	0
Body hydration percentage, mean ± SD	48 ± 6	46 ± 5
General hypermobility^a^, *n* (%)	12 (63)	9 (50)
Composite symptom score (0–96), mean ± SD	47 ± 13	48 ± 12
Evaluation at follow-up		
Malmo POTS Score, mean ± SD	63 ± 16	NA
Quality-of-life (0-100)^a^, median (IQR)	25 (25–50)	NA
Working capacity (0-100)^b^, median (IQR)	0 (0–25)	NA

All variables reflecting prior-to-treatment state. Composite symptom score was derived algorithmically from the standardized questionnaire including 24 questions on typical symptoms associated with ME/CFS.

a *n* = 19 and 16, in completers and non-completers, respectively.

b *n* = 17 and 18, in completers and non-completers, respectively.

None-significant group differences (all *p* > 0.20) if not otherwise stated, calculated from Welch’s *t*-test or Mann–Whitney U test depending on distribution of data.

*p < 0.001 (Welch’s *t*-test).

Additional data on completers (*n* = 22) derived from older diagnostic work-up (performed on average 32 months prior to treatment start) revealed POTS-related symptoms (tilt table test) in 17 (77%) of the patients, while blood chemistry levels were in the normal range for sodium, potassium, glucose, thyroid stimulating hormone (TSH) and cortisol, at that time-point (data not shown).

**Figure 1:**
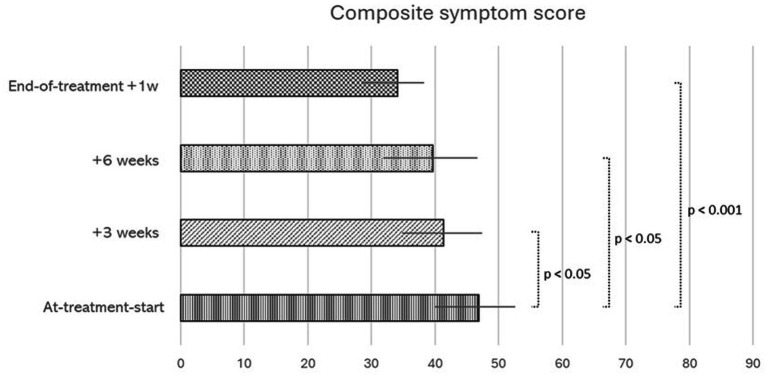
Changes in overall symptom score (mean, SEM) in relation to intermittent infusion of intravenous saline, 3 times over 9 weeks, in patients with ME/CFS who completed the treatment regime: (i) At-treatment-start, prior to first infusion (*n* = 22) (ii) + 3 weeks, prior to the second infusion (*n* = 21), (iii) + 6 weeks, prior to third and last infusion. (*n* = 21), and (iv) End-of-treatment, 1 week after last infusion (*n* = 19) N differs due to incomplete questionnaire response. Scores are derived from a questionnaire administered to patients prior to each treatment session. Including the 24 most prevalent symptoms in ME/CFS. The composite score ranges from 0 to 96, with 96 representing worst possible overall symptoms. *p*-values from Wilcoxon signed rank test.

Among completers, the mean infusion volume over the three treatment occasions was 1,600 (±360) ml and the corresponding mean weight gain at each treatment was 1.4 (±0.5) kg. Mean physiological changes during treatment sessions (pre- to post infusion) revealed a significant decrease in heart rate (−11 bpm, SD 10; *p* = 0.02) by saline infusion, while SBP and DBP were unchanged. Symptoms were monitored during the 9-w treatment regime, i.e., prior to each infusion session as well as 1 week after treatment ended. Changes in overall symptom are depicted in Figure 1, showing successive improvement in the composite symptom score throughout the treatment period. The mean composite symptom score decreased from 47 ± 13 at treatment start, to 34 ± 11 at the end of treatment (*p* < 0.001), on a 0–96 scale. Individual symptoms changed accordingly.

**Figure 2:**
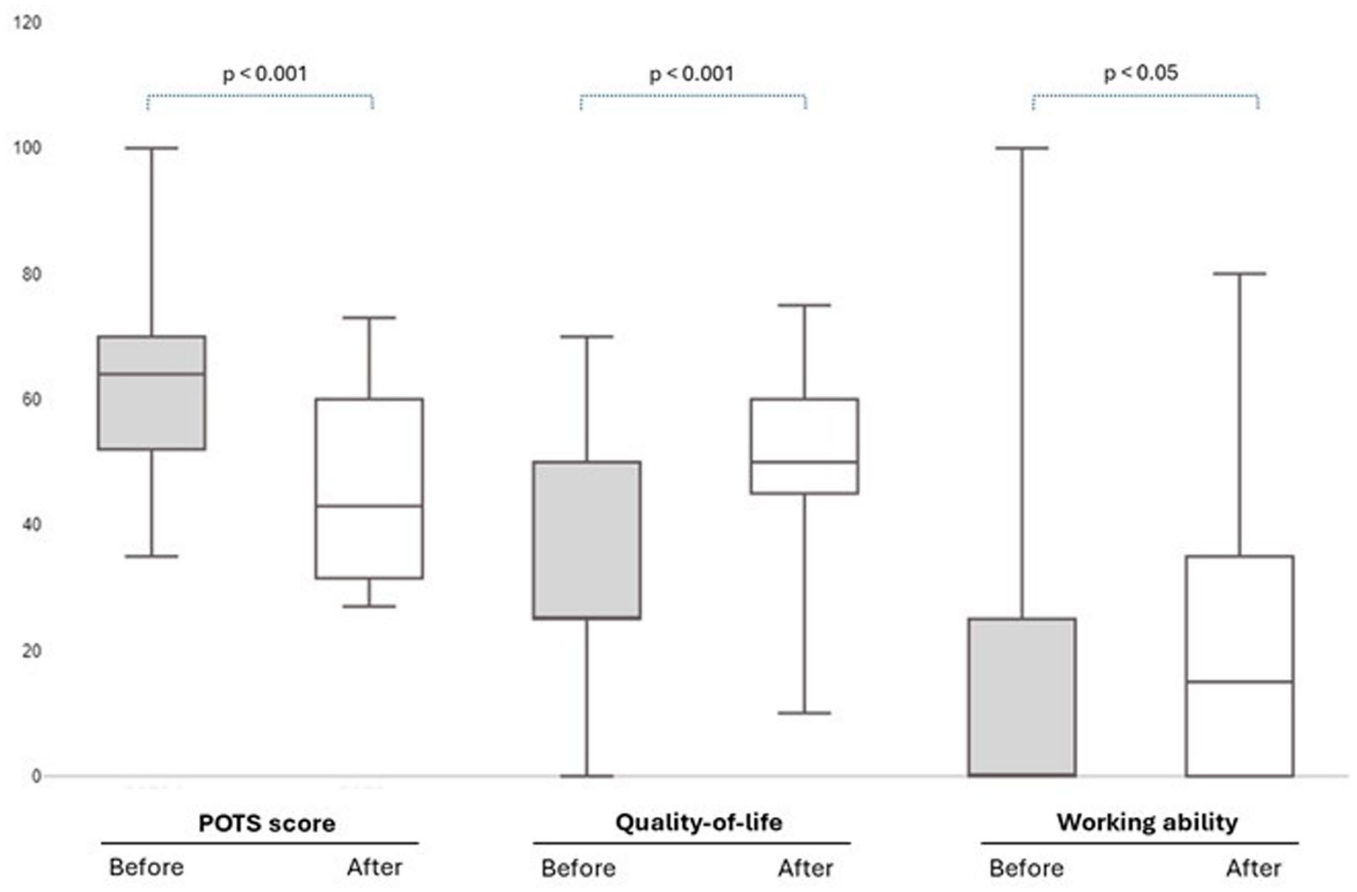
Self-reported changes in POTS score, Quality-of-life and Working ability Before (At-treatment-start) and After (1 week after treatment stop) intermittent infusion of intravenous saline over 9 weeks, in 17 patients with ME/CFS who completed the treatment regime as well as the follow-up questionnaire. The POTS score could take a value of maximum 120, and Quality-of-life and Working ability could take maximum values of 100. *p*-values from Wilcoxon signed rank test.

Among 19 responders at follow-up, the mean subjective benefit of the treatment was 5.5 ± 2.4 on a 0–10 scale, 8 (42%) wanted to continue the infusions, 10 (53%) were unsure, whereas 1 (5%) did not want to continue. As depicted in Figure 2, the median POTS score decreased from a pre-treatment level of 64 (IQR 16) to a post-treatment level of 43 (IQR 29), on a 0–120 scale (*p* < 0.001). Quality-of-life increased from a pre-treatment level of 25 (IQR 25) to a post-treatment level of 50 (IQR 15), on a 0–100 scale (*p* < 0.001), and median working ability increased from a pre-treatment level of 0 (IQR 25) to a post-treatment level of 15 (IQR 35), on a 0–100 scale (*p* < 0.05). Among those who reported the highest benefit of the treatment (≥6 on a scale 0–10, *n* = 8), QoL increased with 29 units on a 0–100 scale, ability to work increased with 14 units on a 0–100 scale, and the POTS score decreased with 26 points on a 0–120 scale. These individuals all had general joint hypermobility and had numerically lower hydration levels at baseline (44 ± 5%), as compared to patients who reported no benefit of the treatment (52 ± 6%), *p* = 0.22 with a small sample size of 7.

The infusions were generally well tolerated. No apparent infusion-related serious side-effects were reported. The majority of the patients (*n* = 22, 55%) attended all three sessions, and most of those (*n* = 19, 86%) completed the follow-up questionnaire. 18 (45%) of the 40 originally included discontinued or missed out on one or two treatment sessions. One patient reported a treatment-related decrease in quality-of-life and working ability, but none of the patients reported worsening of their POTS symptoms. 17 of our 22 patients completed a new TILT-test on average 9 days after the final infusion (range 5–14 days). All 17 had POTS-related symptoms at this occasion with an average increase in heart rate of 37 ± 15 bpm, as compared to a corresponding increase of 35 ± 14 bpm at tilt table testing 6-to-70 months earlier. The self-reported mean effort to comply with the treatment regime, including traveling back-and-forth to the clinic, was 4.6 ± 2.2 on a 0–10 scale with the maximum being 8. Fourteen individuals (64%) reported PEM in relation to clinical visits, and one of those reported severe PEM.

## Discussion

Data from this non-controlled case-series indicate health benefits from volume loading with intermittent infusion of saline among patients with dysautonomia and ME/CFS. QoL and ability to work improved significantly following three treatments over 9 weeks, and an overall improvement in a range of individual symptoms was evident, especially symptoms related to POTS/dysautonomia. These results provide a justification for further work to determine the optimal target group, frequency and methods, for volume loading in patients with ME/CFS who suffer from debilitating dysautonomia.

To our knowledge, no previous studies have investigated IV volume loading as a therapeutic mean in patients suffering from ME/CFS. Our results showing substantial beneficial effects of IV volume loading in a subgroup of patients with ME/CFS are consistent with those of Ruzieh et al., who reported substantial improvements of symptoms in patients with refractory POTS, with a similar treatment regime (21). In that study, general hypermobility predicted poor outcome of IV saline infusion, contrary to the results in our study population. In fact, patients who reported the highest benefit from saline infusions all had general hypermobility. General hypermobility is over-represented in patients with ME/CFS (24), but how dysfunctional connective tissue affects susceptibility for ME/CFS is currently unclear.

The beneficial effects observed in our study are most likely explained by volume loading counteracting existing hypovolemia, as mean body hydration percentage prior to treatment-start was 48 ± 6% in the 22 completers, and as low as 44 ± 5% in those (*n* = 8) who reported the highest benefit of the treatment. We were, however, unable to detect any significant changes in hydration percentage over the treatment period, which might be explained by the insensitive methodology applied (BIA) prohibiting intra-individual conclusions. Possibly other factors may have contributed to the observed orthostatic improvements, such as improved hemodynamics as indicated by volume loading influencing HR in our individuals. Volume loading per se has shown to improve cardiac output, stroke volume and decrease total vascular resistance (17), and the combination of hypovolemia, reduced capillary flow and capillary stasis has been proposed as a strong mediator of impaired organ perfusion and symptomatology in afflicted individuals (25). Why hypovolemia develops in certain individuals is presently unclear but may partly be explained by disruptions in the secretion of pituitary vasopressin, i.e., antidiuretic hormone, regulating body fluid balance. We recently detected non-physiological levels of vasopressin levels despite high serum osmolality in 111 consecutive patients with ME/CFS following overnight fasting and fluid deprivation (26), lending support to previous studies concluding disrupted vasopressin secretion in this patient group (27, 28). Dysregulation of vasopressin release may be one mechanistic explanation for hypovolemia in ME/CFS with its debilitating symptoms and thus represent a potential target for treatment.

The acute beneficial effects of volume loading in POTS and OI-related conditions are well described in the literature (18), but how to attain more sustainable effects with IV volume loading remains an open question. Long-term infusion of intravenous saline is not recommended as clinical trials are lacking and may further be compromised by the need for chronic central venous catheter with its attendant complications (7). Whether some variant of intermittent volume loading can yield sustainable health effects remains to be shown. In the present study, we were not able to detect any sustainable effects and with limited improvements in objective measures. For example, POTS diagnosis was unaffected by intermittent volume loading. This may be due to the time-delay (on average 9 days) between the last infusion and POTS assessment and the results from tilt table test may had been different if performed closer to the infusion session. However, subjective benefits were present among many of our patients and according to previous publications, volume loading does not necessarily have to be concomitant with more objective measures (29). Improvements in subjective measures may increase the patient’s compliance to other interventions with a feed-forward mechanism (19), rendering volume loading as a potentially important bridge therapy for patients suffering from dysautonomia and ME/CFS. Some of the beneficial effects in our study were counteracted by the effort that came with visiting the clinic, which, not unexpectedly, gave rise to PEM in many of our patients. However, it is interesting to note that among our patients, all with severe degree of ME/CFS (*n* = 5) completed the infusion protocol, indicating that disease severity does not influence the capacity to complete this particular treatment.

Patients included in this treatment-series did not deviate substantially from regular patients with ME/CFS at the clinic considering age, gender distribution, disease severity, health status and hypermobility (data not shown). All patients displayed clinical signs of hypovolemia and/or dysautonomia prior to inclusion, but not all improved by the treatment. Hypothetically, using a different solution, different infusion frequency, or longer treatment duration could have generated better and/or more sustainable effects. Our provisional results in this small series will need to be confirmed in a randomized, blinded trial. Future intervention studies could evaluate the effects of volume loading providing precalculated inadequate vs. adequate volumes of saline with a blinded design. Such a study would benefit from using a more sensitive technique to measure body composition and body fluid balance, as compared to the BIA-methodology used in the present study. Finally, side-effects of our treatment protocol were few and harmless.

In summary, we find promising effects on ME/CFS symptomatology from intermittent IV infusion of saline among ME/CFS cases with dysautonomia/hypovolemia. The findings from this case-series may stimulate further studies on various forms of IV volume loading, with or without attendant medications, aiming to reverse ME/CFS symptomatology with sustainable long-term effects.

## Data Availability

The raw data supporting the conclusions of this article will be made available by the authors, without undue reservation.
